# 

**Published:** 1880-04

**Authors:** 


### THE BISTOURY:

Thad S. Up de Graff, M. D., Editor & Proprietor.

### CONTENTS FOR APRIL, 1880.

**CONTRIBUTIONS.** PAGE.

The Air We Breathe,.......BEN. H. PRATT,........ 226
Honored Local Names,....AUSBURN TOWNER........ 229
My School Teacher,........J. J. KEYES........ 231
Old Age,..................THOS. K. BEECHER........ 235
The Monument to Adam,..J. B. CHANDLER........ 237

**MEDICAL ITEMS AND NEWS.**

Nausea—Quinia and Iron Tonic—Cracked Nipples—Tasteless Saline Purgatives—Prostatic Enlargement—Diabetes—Ergot in Catarrhal Affections—Neuralgia—Ovarian Dismenorrhœa—Nausea of Pregnancy—Painful Sores—Precaution in Administering Iron—Sea-Sickness—Cystitis in the Male—Antiscorbutic Tincture—Make Leeches Bite—Whooping Cough—Eczema Intertrigo of Infants—Prevention of Mammary Abcess—Simple Glaucoma—Scabies—Dog Fennel—Habitual Constipation—Viseral Disorders—Acute Coryza—Urticaria—Fissured Nipples—Fluid Extract Grindelia Squarrosa—Obesity—Diphtheria—Trichinæ—Blistering for Neuralgia—Diarrhœa—Glaucoma—Diseases of Bladder—Quinine for Children—Anti-Neuralgic—Intermittent Fever—Diarrhœa in Tuberculous Patients—Rosacea of Face—New Anthelmintic—Whooping Cough—Gargle the Throat—Vaginismus—Hypodermic Syringe—Hemorrhage of Umbilicus in Infants—Supra-orbital Neuralgia Cured by Nerve-stretching—Goitre—Milk Diet for Articular Rheumatism—Bloodless Method of Operating—Urticaria..........238 to 247

**EDITORIAL.**

SCHOOL HYGIENE—I........ 247
INHUMATION........ 249
THE AUDIPHONE........ 250

**CHIT-CHAT.**

In the Woods—Not Half—Eldridge Park—Stop it—The Contributions—Daily Advertiser—That Home—The Telephone—Frits Gets Tired of Being Good—The Monument........251 to 254

